# Ontogenetic trophic segregation between two threatened smooth-hound sharks in the Central Mediterranean Sea

**DOI:** 10.1038/s41598-020-67858-x

**Published:** 2020-07-03

**Authors:** Manfredi Di Lorenzo, Salvatrice Vizzini, Geraldina Signa, Cristina Andolina, Gabriele Boscolo Palo, Michele Gristina, Carlotta Mazzoldi, Francesco Colloca

**Affiliations:** 1 Institute for Biological Resources and Marine Biotechnologies, National Research Council (IRBIM-CNR), Via Luigi Vaccara 61, 91026 Mazara del Vallo, TP Italy; 2 CoNISMa, National Interuniversity Consortium for Marine Science, Piazzale Flaminio 9, 00196 Rome, Italy; 3 Department of Earth and Marine Sciences, University of Palermo, Via Archirafi 18, 90123 Palermo, Italy; 4 Department of Biology, University of Padova, Via Ugo Bassi 58/B, 35131 Padua, Italy; 5 Institute of Anthropic Impacts and Sustainability in Marine Environment, National Research Council (IAS-CNR), Via G. da Verrazzano, 17, 91014 Castellammare Del Golfo, TP Italy; 6 Anton Dohrn Zoological Station, Integrative Marine Ecology Department, Via Po 25, 00198 Rome, Italy; 7 Department of Biology and Biotechnology “Charles Darwin”, Sapienza University of Rome, Rome, Italy

**Keywords:** Behavioural ecology, Stable isotope analysis

## Abstract

Elasmobranchs are among the species most threatened by overfishing and a large body of evidence reports their decline around the world. As they are large predators occupying the highest levels of marine food webs, their removal can alter the trophic web dynamic through predatory release effects and trophic cascade. Suitable management of threatened shark species requires a good understanding of their behaviour and feeding ecology. In this study we provide one of the first assessments of the trophic ecology of the “vulnerable” smooth-hounds *Mustelus mustelus* and *M. punctulatus* in the Central Mediterranean Sea, based on stomach contents and stable isotope analyses. Ontogenetic diet changes were addressed by comparing the feeding habits of three groups of individuals: juveniles, maturing and adults. Our results highlighted that the two species share a similar diet based mostly on the consumption of benthic crustaceans (e.g. hermit crabs). Their trophic level increases during ontogeny, with adults increasing their consumption of large-sized crustaceans (e.g. *Calappa granulata*,* Palinurus elephas*), cephalopods (e.g. *Octopus vulgaris*) and fish (e.g. *Trachurus trachurus*). Our results provide also evidence of ontogenetic shifts in diet for both species showing a progressive reduction of interspecific trophic overlap during growth. The results of this study contribute to improve the current knowledge on the trophic ecology of these two threatened sharks in the Strait of Sicily, thus providing a better understanding of their role in the food web.

## Introduction

Elasmobranch species are commonly recognized to be important predators in the marine realm^1^ and play a crucial role in regulating marine ecosystems^2,3^. Greater awareness of the trophic ecology of sharks can provide important information about the role they play during their life cycles and improve understanding of marine communities’ structure and functioning. Many decades of severe human impacts have led to a rapid decline of many elasmobranch species around the world^4,5^, exacerbated by their biological vulnerability (e.g. slow growth rate, low fecundity, and late age at maturity)^2,5^. As a consequence, many shark species are now registered by the IUCN as threatened or endangered^6^. It has been shown that the decline of elasmobranch species has had marked ecological consequences^7,8^. The loss of predators may negatively alter the food chain, triggering new interactions among species and marine ecosystem degradation^9^. Therefore, improved knowledge about the elasmobranch trophic ecology, including the prey consumed, trophic level, ontogenetic diet changes, especially in the Mediterranean species, can play a crucial role in the development of new fishery management strategies. In fact, resource partitioning is one of the main processes for the co-existence of species^10^. Partitioning, which has been observed in several organisms^12−14^, can occur along the space, time, or feeding niche axes^11^, during the same or at different ontogenetic stages. In this way, different species can use similar feeding resources, which decreases diffuse competition, and allows the coexistence of species within a given ecosystem^15−17^. Variability in trophic spectrum may lead to coexistence of several species inhabiting the same area through the exploitation of different prey items^18^.

The genus *Mustelus* (Linck, 1,790) of the family Triakidae in the order Carcharhiniformes includes about 27 valid extant species in the world’s oceans^19^. It is probably the most challenging group of elasmobranchs concerning taxonomic aspects because species are often difficult to identify due to their conservative morphology, combined with highly intraspecific variable diagnostic characteristics^20^. *Mustelus* species, commonly known as smooth-hounds, have a high level of regional endemicity and are commonly distributed on the continental shelf in temperate to tropical waters^21,22^. They are small to medium-sized demersal mesopredator sharks commonly exploited as target species or caught as by-catch by several different types of fisheries (e.g. artisanal, trawlers, recreational) in many oceanic areas^2,23^. Smooth-hounds are all viviparous, with reproductive modes including both placental and yolk-sac viviparity^20^. Some studies have also shown that smooth-hounds and other mesopredator sharks have an important role in marine ecosystems, inducing top-down control on the populations of their prey when their abundance is not consistently reduced by apex predators, such as big sharks^7,24^ or by fishing.

In the Mediterranean Sea, there are three smooth-hound species: the starry smooth-hound, *Mustelus asterias* Cloquet, 1819, the common smooth-hound, *M. mustelus* (Linnaeus 1758), and the blackspotted smooth-hound, *M. punctulatus* (Risso, 1827)^20^. The last two species, inhabiting the shelf up to − 200 m deep^25,26^, are morphologically very similar and consequently often misidentified during field work. More so because identification guides or papers report different and sometimes contrasting diagnostic traits. Such species similarity might be the result of a relatively recent speciation event and it is not surprising that in a recent genetic study, the authors found signs of hybridisation events between *M. mustelus* and *M. punctulatus*, although hybrid viability is actually unknown^27^. On the other hand, the starry smooth‐hound is more easily distinguishable because of its body colouration and evident white spots^28^. Sharing the same area and having similar morphological characteristics, these species could compete for food resources. All the three smooth-hounds are threatened in the Mediterranean region. According to the IUCN's European Red List of marine fish^6^, *M. mustelus* and *M. punctulatus* are classified as ‘Vulnerable’ and *M. asterias* is evaluated as ‘Near Threatened’. However, a recent study has shown a decline in common and blackspotted smooth‐hounds of 72% and 78% respectively over three generations (i.e. 60 years), thus suggesting that in the next regional assessment a shift to the ‘Endangered’ category should be considered^29^. The case of the starry smooth-hound is even worse since it has disappeared from most of the Mediterranean coastal areas suggesting a high risk of regional extinction^29^. The current smooth-hound status is the result of a long fishing exploitation history that began in the neolithic and has rapidly accelerated over the past 50 years due to the expansion of trawling^30,31^. Currently, smooth-hounds are still exploited or caught as by-catch in trawling and artisanal fisheries in the few Mediterranean areas where viable populations are still existing, such as Tunisia^32^, Northern Adriatic^33,34^ and Mediterranean Turkish coasts^26,35,36^.

In this study we focused on the trophic ecology of the common (*M. mustelus*) and blackspotted smooth-hound (*M. punctulatus*) in the Northern sector of the Strait of Sicily (SoS), an area that probably hosts some of the last viable populations of these species in European Mediterranean waters^29^.

The knowledge about life history traits of the two species is poor and geographically scattered. Information on sexual maturity and reproduction is available for Tunisia^25,32,37,38^ and the Northern Adriatic Sea^33^. Age and growth data are available only for the common smooth-hound in the northern Aegean Sea where it appears late-maturing, long-lived and slow-growing^26^. Similarly, current knowledge about prey preferences is limited to studies conducted in the Northern Adriatic for the black spotted smooth-hound, in the Western Mediterranean (Gulf of Valencia, Spain) and Aegean Sea for the common smooth-hound^37,39^ and in Tunisia for both species^25,40^. All these studies showed that the smooth-hounds are important predators of coastal crustaceans, cephalopods and fish, and may also have a major impact on important commercial species, such as the spiny lobster and common octopus, with potential repercussions for coastal fisheries.

The main objective of our study is to elucidate the trophic role played by the common and the black-spotted smooth-hounds in the SoS for a better understanding of the possible factors triggering species coexistence. To this aim, diet composition, trophic niches and trophic levels were analysed across ontogenetic stages through a combined approach based on stomach contents and stable isotope analysis.

## Material and methods

### Study area

**Figure 1:**
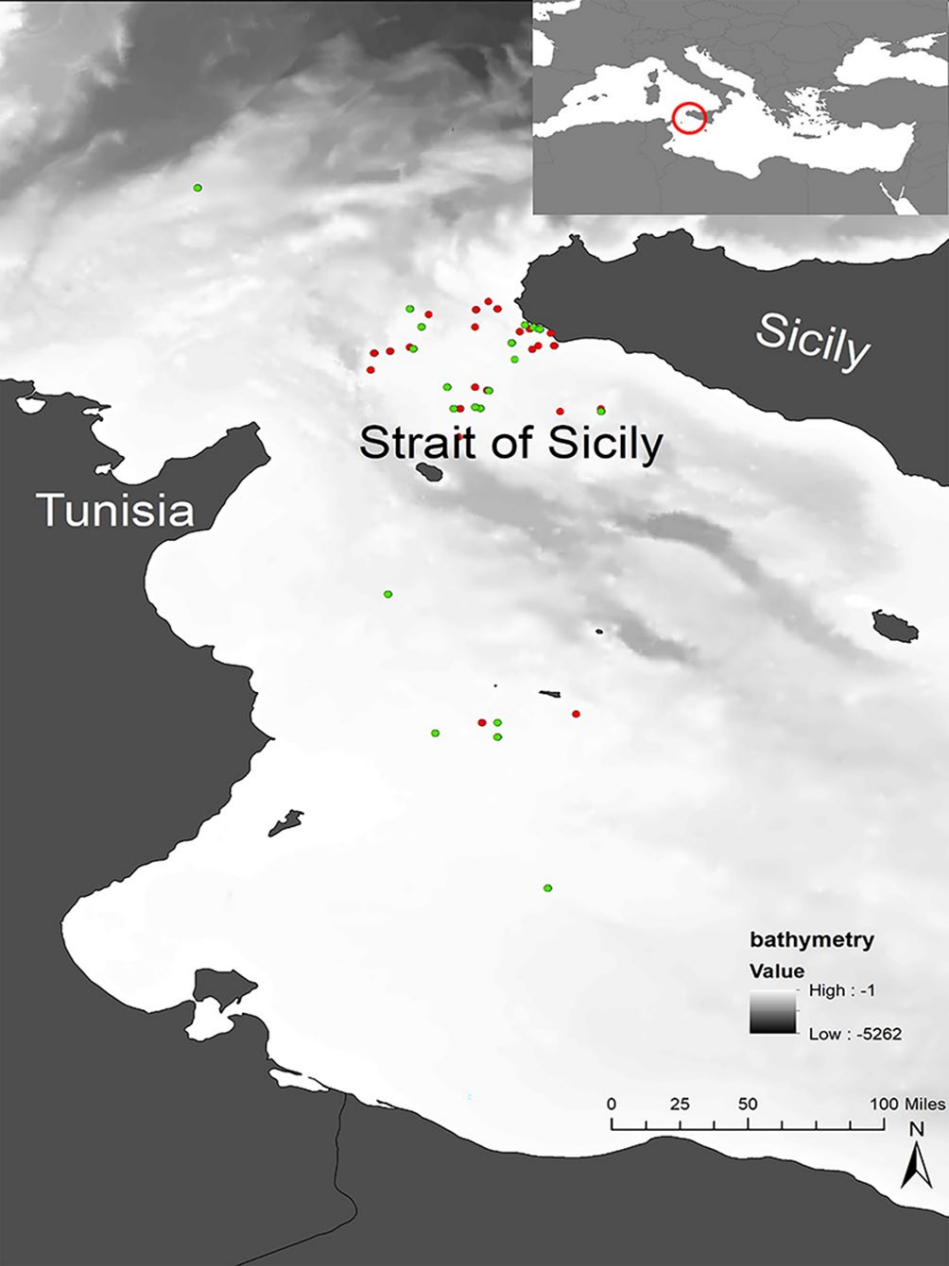
Study area, Strait of Sicily (SoS). Red and green points indicate the capture sites of *Mustelus mustelus* and *Mustelus punctulatus* respectively. Red and green are often overlapped. This map was created with ArcGIS version 10.6.1 https://www.arcgis.com/index.html# by MDL.

The study area covers approximately 34.000 km^2^ off the southern coast of Sicily, corresponding to the North sector of the Strait of Sicily (SoS—Fig. 1, see^41^). It is characterised by high primary productivity and high levels of biodiversity due to the occurrence of complex and diverse benthic biocoenosis^42,43^. Recent studies showed high diversity and biomass of demersal communities over the offshore detritic bottoms of the Adventure bank^43−45^. Given the importance of this area for conservation, several sites have been identified for inclusion in a Mediterranean network of marine protected areas^46^. The off-shore banks are particularly important for elasmobranchs both in terms of species diversity and abundance^48^. In addition, the SoS is one of the most important Mediterranean fishing areas, with a high concentration of fishing vessels from different countries (i.e. Italy, Malta, Tunisia), which exploit a wide range of demersal and pelagic species^49^. From an oceanographic point of view, the SoS plays a key role for the Mediterranean thermohaline circulation^50^ and the exchange of water masses between the eastern and western Mediterranean basins. The Atlantic Ionian Stream (AIS) and the Atlantic Tunisian Current (ATC) flow across the SoS^51^. The AIS is associated with a number of well-known semi-permanent features in particular two large cyclonic vortexes; the first one lies over the Adventure Bank and the second, over the Malta shelf, off Capo Passero.

### Shark sample collection

All procedures carried out were approved by the international authorities (EU/DG Mare, FAO/GFCM). All methods were performed in accordance with the relevant guidelines and regulations. In the cases the animal was alive when it arrived on the vessel during the scientific survey (MEDITS—DCF, EU Reg. 199/2008), it was suppressed by administering an overdose of anaesthetic in compliance with the recommendation of Decree Law n. 26 of 4 March 2014. All efforts were made to minimize suffering.

Individuals of *M. mustelus* (mmus) and *M. punctulatus* (mpun) were gathered both from local fishing fleets, i.e. trawlers and artisanal vessels that use trammel nets, gillnets and longlines, and during the MEDITS bottom trawl surveys carried out in the North sector of the Strait of Sicily (FAO-GFCM, GSA 16) in the summer of the years 2013, 2015, 2016 and 2017. The temporal range of sampling was necessarily wide to collect an adequate number of samples to describe the diet composition of such poorly abundant species.

Specimens were immediately placed on ice after capture and taken to the laboratory. Once in the laboratory, sharks were identified, measured to the nearest 1 cm (total length, TL), weighed to the nearest 1 g, and both sex and maturity were registered using the maturity scale for viviparous sharks adopted by the MEDITS survey protocol^52^. Species identification was based on the inspection of dermal denticles using a stereomicroscope^53^. After measurements, stomachs were removed and stored in labelled plastic boxes filled with a 70% ethanol solution. In addition, from the individuals caught during the last sampling year (2017), a small section of dorsal muscle (1 g) of both *M. mustelus* (n = 58: 36 juveniles, 18 maturing, 4 adults) and *M. punctulatus* specimens (n = 39: 20 juveniles, 14 maturing, 5 adults) was removed for stable isotope analysis, rinsed with deionised water, oven dried for 3 days at 60 °C, then ground with a pestle and mortar and kept in a dry room until analysis.

### Gut content analysis

In order to assess dietary ontogenetic shift of the two *Mustelus* species, the specimens of each species were grouped in three size classes according to sexual maturity: juvenile (mmus: TL ≤ 80 cm; mpun: TL ≤ 70 cm); maturing (mmus: 81–105 cm TL; mpun: 71–90 cm); and adult (mmus: TL > 105 cm; mpun: TL > 90 cm)^25,38^. To obtain a precise description of the diet, it is important to determine the minimum number of stomachs required (Ferry and Cailliet 1996). The number of both mmus and mpun collected was tested to determine whether sufficient specimens were sampled. Given the rather low number of stomach contents, especially for adults, we decided to pool together the available stomach sample data and calculate the saturation curves by species. The cumulative number of randomly pooled stomachs was plotted against the cumulative number of prey taxa.

Prey items in each stomach were sorted and determined to the lowest possible taxonomic level. For a more accurate prey identification, all the hard parts were cleaned with distilled water and otoliths were identified using otolith Atlas^47^. Then, all prey items were counted and weighed. For the evaluation of the diet composition of each species and size class, we calculated the following indices: (1) Vacuity Index (VI = number of empty stomachs/total number of stomachs × 100), (2) relative frequency of occurrence (%*F* = number of stomachs containing prey *i*/total number of full stomachs × 100), (3) relative numerical abundance (%*N* = number of prey *i*/total number of prey × 100), and (4) relative gravimetric composition (%*W* = weight of prey *i*/total weight of all prey × 100). The index of relative importance (*IRI*) of^54^, as modified by^55^, was used to assess the importance of each prey item in the diet of the two species at each size class: *IRI* = %*F* × (%*N* + %W). Prey species were listed in decreasing order according to their relative *IRI* contribution and then *%IRI* was calculated for each prey item as follows:

$$\% IRI = \left( {IRI_{i} / \sum \limits_{i}^{N} IRI_{i} } \right) \times 100$$

Interspecific differences in diet composition were tested using permutational multivariate analysis of variance (PERMANOVA)^56^ and based on the prey biomass from the Bray Curtis similarity matrix were obtained from square root transformed data.

The analysis, carried out with the PRIMER statistical package 6.0^57^, included one factor: “species” (two levels: mmus and mpun).

The effect of year on the diet composition of both species size classes was not analysed due to the limited number of samples available, thus implicitly assuming a constant diet through time.

### Stable isotope analysis

Subsamples of dorsal muscles destined to carbon stable isotope analysis were delipidated before conducting analysis. The lipids were removed by rinsing the ground tissue several times with 2:1 chloroform:methanol mixture according to^58−60^. For both species and all size classes, approximately 1 mg of individual muscle sample was weighed in tin capsules, automatically loaded in an elemental analyser (Thermo Flash EA 1,112) for the determination of total carbon and nitrogen, and then analysed for *δ*^13^C (from delipidated subsamples) and *δ*^15^N (from bulk subsamples) in a continuous-flow isotope-ratio mass spectrometer (Thermo Delta Plus XP). Stable isotope ratio was expressed, in relation to reference international standards (atmospheric N_2_ and PeeDee Belemnite for *δ*^15^N and *δ*^13^C respectively), as:

$$\delta^{13} {\text{C}}\,{\text{or}}\,\delta^{15} {\text{N}}\,\left( \permil \right) = \left[ {\left( {{\text{R}}_{{{\text{sample}}}} /{\text{R}}_{{{\text{standard}}}} } \right) - 1} \right] \times 10^{3}$$

where R is the ^13^C/^12^C or ^15^ N/^14^ N respectively. Analytical precision based on standard deviations of internal standards (International Atomic Energy Agency IAEA-CH-6; IAEA-NO-3; IAEA-N-2) ranged from 0.10 to 0.19‰ for *δ*^13^C and 0.02 to 0.08‰ for *δ*^15^N.

The changes in carbon and nitrogen stable isotopic signatures of the two *Mustelus* species with size increasing was evaluated through linear regressions between *δ*^13^C (‰) and *δ*^15^N (‰) as dependent variables and size (TL, cm) as the independent variable. To test for differences in isotopic niche width between the two *Mustelus* species and among size classes, standard ellipse areas (SEAc and SEAb) were estimated by Bayesian statistics based on carbon and nitrogen stable isotope data. In more detail, SEAc, which is the standard ellipse area corrected for small sample size, was set to contain 40% of the data and provided unique values, while SEAb, which is the Bayesian standard ellipse area, derives from 4,000 posterior iterations and is reported as mode along with 95% credible interval^61^. Differences in SEAb between species and among size classes were tested through pair-wise comparisons, by calculating the probability that the SEAb of one group is larger than that of the other, hence a significant difference was regarded as a probability of at least 95%^61^. SEAc, SEAb, as well as the overlap between SEAc, were estimated with R package SIBER v. 2.1.3 (Stable Isotope Bayesian Ellipses in R)^61,62^.

Trophic positions based on stable isotope data (TP_SIA_) were estimated according to the following equation^63^:

$${\text{TP}}_{{{\text{SIA}}}} = \left[ {\left( {\delta^{15} {\text{N}}{-}\delta^{15} {\text{N}}_{{\text{b}}} } \right)/\Delta_{n} } \right] + \lambda$$

where *δ*^15^N and *δ*^15^N_b_ are respectively the nitrogen isotopic signature of each *Mustelus* specimen, and that of the baseline, for which we used the mean value of *Pagurus prideaux* (see results) sampled in the same study area (6.88 ± 0.36 ‰). *Δ*_n_ is the trophic enrichment expected for each trophic level (2.78 ‰), according to^64^, and λ is the trophic position of the baseline, that was set to 3 as the mean in the literature data^65,66^. Additionally, trophic position for the three size classes of both target species were calculated based on stomach content data (TP_SCA_) following the method proposed by^67^:

$${\text{TP}}_{{{\text{SCA}}}} = 1 + \left( {\sum {\text{P}}_{j} \times {\text{TL}}e_{j} } \right)$$

where TL*ej* is the trophic level of each prey category *j* and P*j* is the proportion of each prey category to the diet of the species. Prey categories taken into account were cephalopoda, decapoda, fish, molluscs and other invertebrates, whose relative trophic level was taken from^67^.

## Results

**Figure 2:**
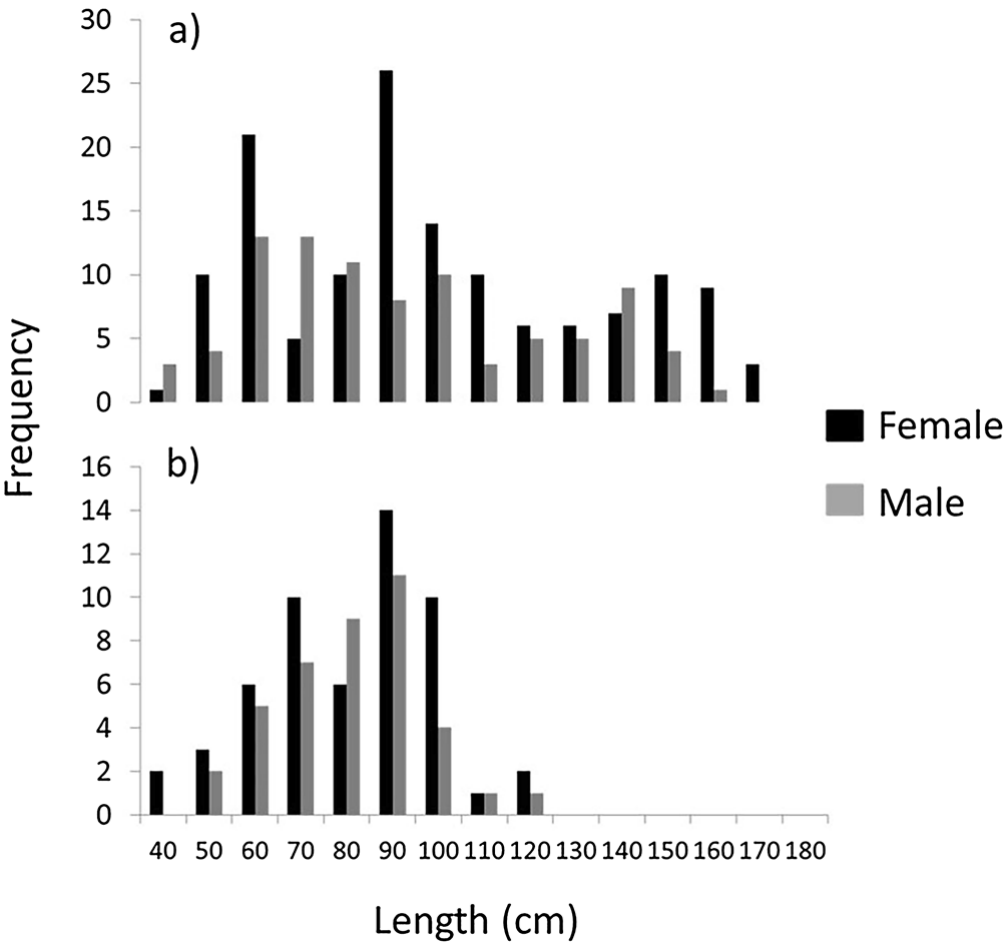
Length-frequency distributions of (**a**) *M. mustelus* (n = 234) and (**b**) *M. punctulatus* (n = 98).

**Figure 3:**
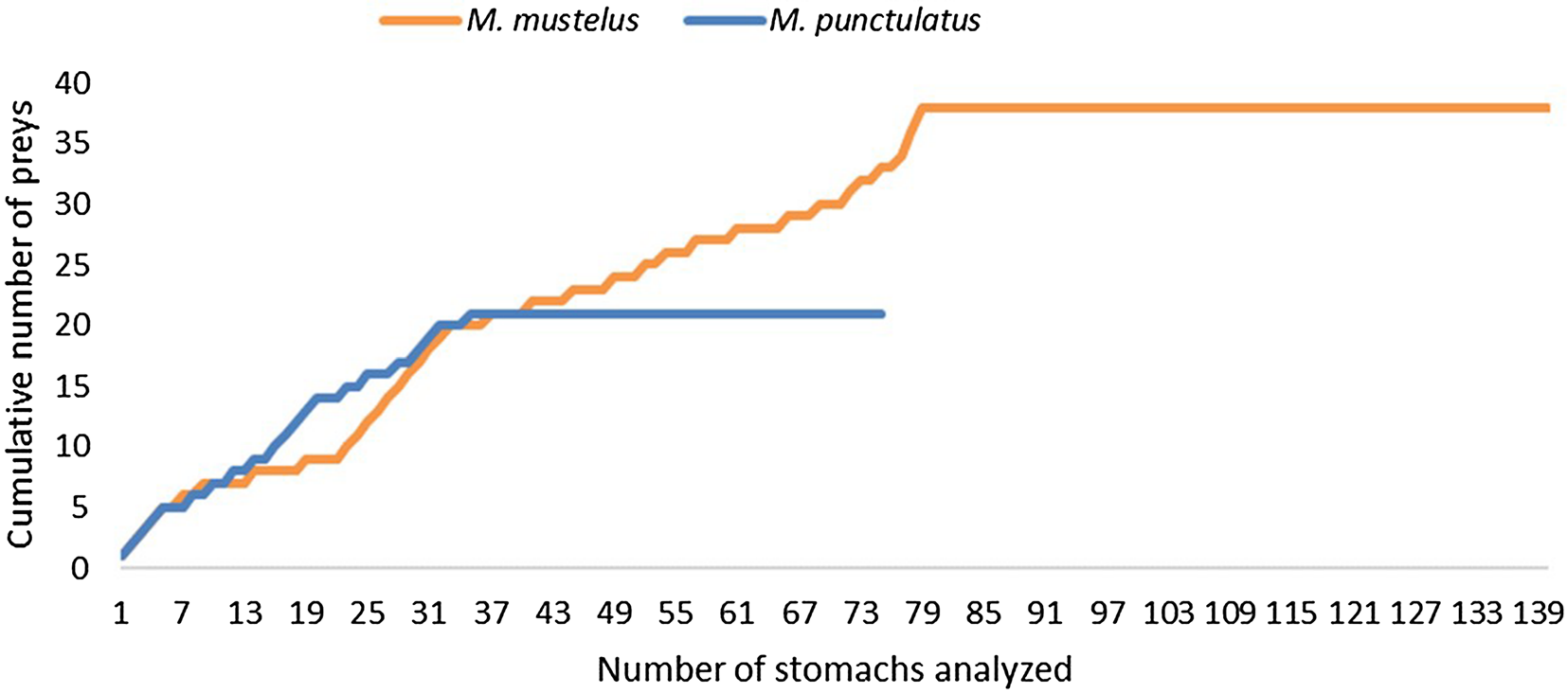
Cumulative prey curves for total number of analysed stomachs of *M. mustelus* and *M. punctulatus.*

We collected 234 specimens of mmus and 98 of mpun, whose sizes ranged between 31.5 and 170.0 cm total length (TL) for mmus, and between 35.5 and 120.0 cm TL for mpun (Fig. 2, Table 1). A higher statistically significant vacuity average index was found in mpun (VI = 34.5%) than in mmus (VI = 23.7%) (Mann–Whitney *p* = 0.0125). The total number of prey taxa found in the stomach was 41 for mmus (some of the prey were found in different size classes) and 27 for mpun (Table 1). The cumulative curves reached an asymptotic value after 55% of analysed stomachs according to mmus and 45% to mpun (Fig. 3).

**Table 1:** Summary of the number of individuals sampled of *Mustelus mustelus* (TL juvenile: ≤ 80 cm; TL maturing: 81–105 cm; TL adult: > 105 cm) and *M. punctulatus* (TL juvenile: ≤ 70 cm; TL maturing: 71–90 cm; TL adult: > 90 cm) collected for the analyses of stomach contents. Some prey taxa were found in more than one size class.

	*M. mustelus*	*M. punctulatus*
Total number	234	98
Juveniles	95	35
Maturing	65	43
Adults	74	19
Females	142	56
Males	92	42
TL range (cm)	31.5–170.0	35.5–120.0
Vacuity Index (%)_Juvenile	19.1	21.4
Vacuity Index (%)_ Maturing	36.9	49.3
Vacuity Index (%)_Adult	15.2	23.0
Number of prey_Juvenile	20	11
Number of prey_Maturing	17	15
Number of prey_Adult	28	5

### Diet composition

**Figure 4:**
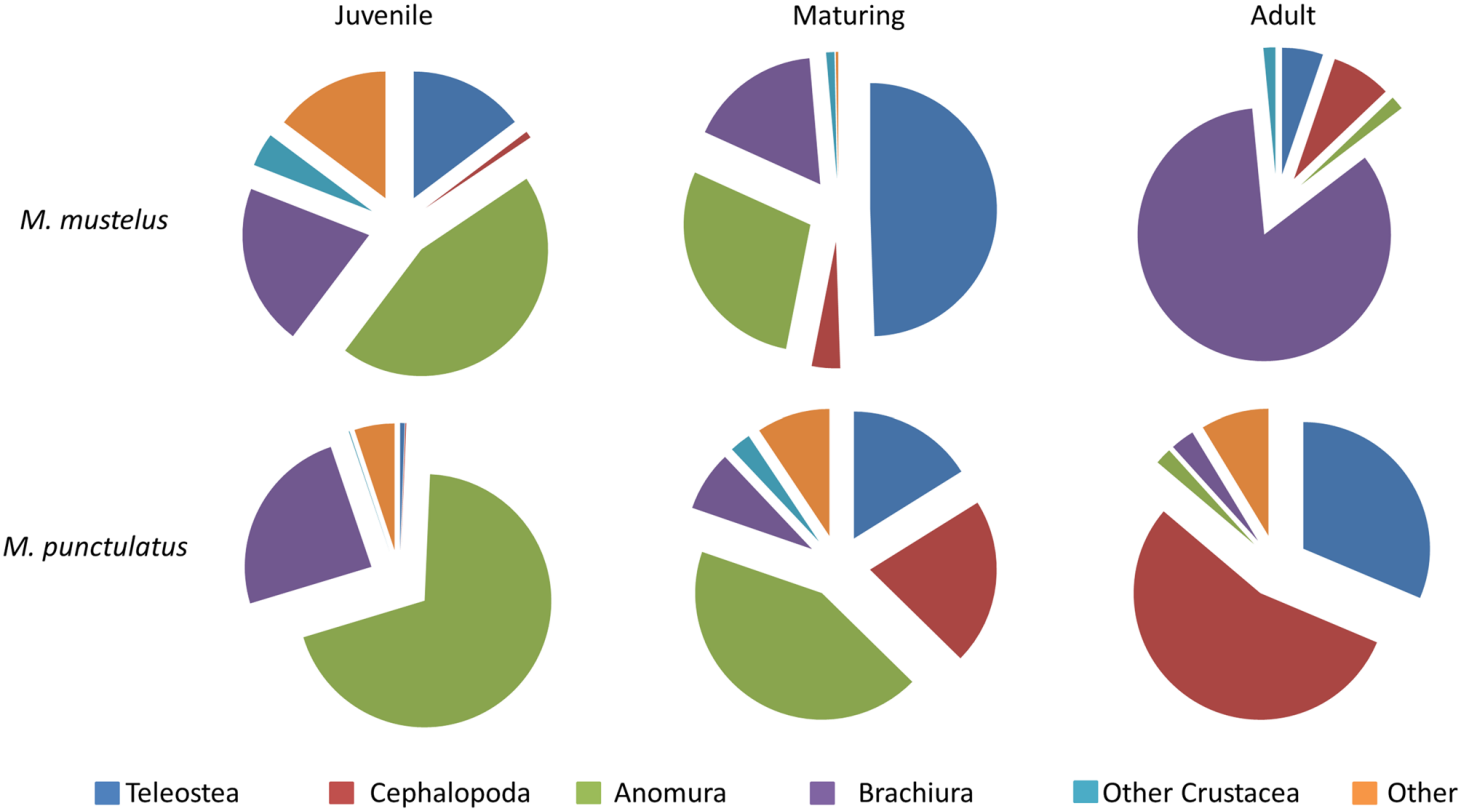
Graphic representation of diet composition, using the percentage of IRI (%IRI), of the three size classes of *M. mustelus* and *M. punctulatus*. This figure was created using Microsoft Excel by MDL.

Crustaceans, mainly Brachiura and Anomura, were the most important prey for mmus juveniles (*%IRI* = 68.6) and adults (*%IRI* = 85.3), while Teleostea were the main prey for maturing specimens (*%IRI* = 49.4) (Table 2; Fig. 4). In more details, juveniles fed mostly upon portunids and hermit crabs, with *Pagurus prideauxi* (*%F* = 13.5; %*IRI* = 29.0) and *Liocarcinus depurator (%F* = 10.9; %*IRI* = 15.6) as the main prey, followed by teleostea as secondary prey (*%IRI* = 14.7). Maturing specimens appeared to switch their diet to fish (*%IRI* = 49.3), which were found generally too highly digested in the stomachs to allow for prey species identification, and Anomura (*%IRI* = 28.6). In this latter group, the most important prey was the hermit crab *P. prideauxi* (*%F* = 11.7; %*IRI* = 27.1), whilst portunids were replaced by the bigger-sized shamefaced crab *Calappa granulata* (*%F* = 8.3; %*IRI* = 12.1). This latter species was dominant in the diet of adult mmus (*%F* = 22.2; %*IRI* = 78.9), which was found to feed also on cephalopods, such as *Octopus vulgaris* (%*IRI* = 5.2) and fish (%*IRI* = 5.0) (Table 2; Fig. 4).

**Table 2:** Diet composition of the three size classes of *Mustelus mustelus* %N: percentage in number; %F: Frequency of occurrence; %W: percentage in mass of prey items; %IRI: percentage index of relative importance. Unid. means Unidentified prey.

Prey	*M. mustelus*_juveniles				*M. mustelus*_maturing				*M. mustelus*_adults			
	F%	N%	W%	%IRI	F%	N%	W%	%IRI	F%	N%	W%	%IRI
Polychaeta unid	1.8	0.9	0.0	0.1	0.0	0.0	0.0	0.0	0.0	0.0	0.0	0.0
Crustacea	61.5	72.8	61.1	68.6	46.7	72.0	40.7	46.5	50.5	68.4	54.2	85.3
***Anomura***												
*Dardanus arrosor*	2.9	2.9	2.6	1.1	0.0	0.0	0.0	0.0	2.0	2.2	0.3	0.3
*Dardanus calidus*	1.0	0.6	0.7	0.1	1.7	1.0	0.1	0.1	1.0	0.7	0.5	0.1
*Pagurus prideauxi*	13.5	19.7	17.9	29.0	11.7	37.0	7.8	27.1	3.0	6.6	1.8	1.3
Anomura unid	11.8	16.8	12.2	14.7	0.0	0.0	0.0	0.0	0.0	0.0	0.0	0.0
***Brachiura***												
*Calappa granulata*	1.0	0.6	1.0	0.1	8.3	2.0	26.0	12.1	22.2	25.0	43.3	78.9
*Dromia personata*	0.0	0.0	0.0	0.0	0.0	0.0	0.0	0.0	1.0	0.7	0.0	0.0
*Galathea strigosa*	0.0	0.0	0.0	0.0	1.7	8.0	1.3	0.8	1.0	0.7	0.1	0.0
*Goneplax rhomboides*	0.0	0.0	0.0	0.0	0.0	0.0	0.0	0.0	1.0	0.7	0.0	0.0
*Liocarcinus corrugatus*	1.0	0.6	0.0	0.0	1.7	1.0	0.5	0.1	1.0	0.7	0.0	0.0
*Liocarcinus depurator*	10.9	13.9	8.0	15.6	6.7	10.0	1.5	4.0	2.0	1.5	0.2	0.2
*Liocarcius vernalis*	1.9	1.7	0.7	0.3	0.0	0.0	0.0	0.0	0.0	0.0	0.0	0.0
*Liocarcinus spp*	5.0	6.9	6.5	4.8	1.7	1.0	0.1	0.1	3.0	5.2	1.3	1.0
*Macroripipus tubercolatus*	1.0	1.2	1.2	0.2	0.0	0.0	0.0	0.0	1.0	7.4	1.9	0.5
*Maja squinado*	1.0	0.6	1.2	0.1	3.3	2.0	1.1	0.5	4.0	2.9	0.8	0.8
*Munida rutllanti*	1.0	1.2	1.3	0.2	3.3	3.0	0.6	0.6	0.0	0.0	0.0	0.0
*Paramola cuvieri*	0.0	0.0	0.0	0.0	0.0	0.0	0.0	0.0	1.0	0.7	0.1	0.0
*Pilumnus hirtellus*	1.0	0.6	0.5	0.1	0.0	0.0	0.0	0.0	1.0	0.7	0.0	0.0
*Palinurus elephas*	0.0	0.0	0.0	0.0	0.0	0.0	0.0	0.0	1.0	0.7	2.5	0.2
*Parapenaeus longirostris*	2.0	2.9	1.5	0.6	3.3	3.0	0.5	0.6	0.0	0.0	0.0	0.0
*Squilla mantis*	2.9	1.7	5.1	1.3	1.7	1.0	0.3	0.1	2.0	1.5	0.9	0.3
*Thia scutellata*	1.0	1.2	0.3	0.1	0.0	0.0	0.0	0.0	0.0	0.0	0.0	0.0
Crostacea unid	2.9	2.3	0.4	0.5	1.7	3.0	0.9	0.3	3.0	10.3	0.4	1.7
Gastropoda unid	0.0	0.0	0.0	0.0	0.0	0.0	0.0	0.0	1.6	0.8	0.1	0.0
*Janthina pallida*	0.0	0.0	0.0	0.0	0.0	0.0	0.0	0.0	1.0	0.7	0.1	0.0
Cephalopoda	1.9	1.7	4.9	1.9	8.3	5.0	28.8	5.8	14.1	11.0	3.0	7.5
*Eledone moschata*	0.0	0.0	0.0	0.0	1.7	1.0	1.8	0.2	3.0	2.9	7.9	1.7
*Octopus vulgaris*	0.0	0.0	0.0	0.0	1.7	1.0	26.0	2.3	5.1	3.7	16.1	5.2
*Sepia* spp	0.0	0.0	0.0	0.0	0.0	0.0	0.0	0.0	1.0	0.7	0.3	0.1
*Alloteuthis sp*	0.0	0.0	0.0	0.0	0.0	0.0	0.0	0.0	1.0	0.7	0.1	0.0
*Todarodes sagittatus*	0.0	0.0	0.0	0.0	0.0	0.0	0.0	0.0	1.0	0.7	2.5	0.2
*Todaropsis eblanae*	0.0	0.0	0.0	0.0	0.0	0.0	0.0	0.0	1.0	0.7	2.9	0.2
Cephalopoda unid	1.9	1.7	4.9	1.9	5.0	3.0	1.1	1.1	2.0	1.5	0.1	0.2
Holothuroidea unid	0.0	0.0	0.0	0.0	1.7	1.0	0.6	2.2	4.8	2.3	1.7	0.4
Teleostea	18.3	12.1	14.6	14.7	40.0	21.0	28.1	49.4	2.0	15.4	8.0	5.0
*Dalophys imberbis*	0.0	0.0	0.0	0.0	1.7	1.0	0.7	0.2	0.0	0.0	0.0	0.0
*Diplodus annularis*	0.0	0.0	0.0	0.0	1.7	1.0	1.7	0.2	1.0	0.7	0.6	0.1
*Diplodus vulgaris*	0.0	0.0	0.0	0.0	1.7	1.0	2.3	0.3	0.0	0.0	0.0	0.0
*Ghathophis mystax*	1.9	2.3	0.8	0.4	0.0	0.0	0.0	0.0	0.0	0.0	0.0	0.0
*Lepidotrigla cavillone*	1.9	1.2	1.5	0.3	0.0	0.0	0.0	0.0	2.0	1.5	0.3	0.2
*Ophisurus serpens*	0.0	0.0	0.0	0.0	0.0	0.0	0.0	0.0	1.0	0.7	0.1	0.0
*Sarda sarda*	0.0	0.0	0.0	0.0	0.0	0.0	0.0	0.0	1.0	0.7	0.3	0.1
*Sardina pilchardus*	0.0	0.0	0.0	0.0	1.7	1.0	1.3	0.2	2.0	1.5	0.3	0.2
*Symphodus tinca*	1.0	0.6	1.0	0.1	0.0	0.0	0.0	0.0	0.0	0.0	0.0	0.0
*Symphodus* spp	0.0	0.0	0.0	0.0	0.0	0.0	0.0	0.0	1.0	0.7	1.8	0.1
*Trachurus trachurus*	1.9	1.2	0.6	0.2	3.3	3.0	5.9	1.5	1.0	5.2	2.1	0.4
Teleostea unid	11.5	6.9	10.7	13.7	30.0	14.0	16.2	47.0	11.1	4.4	2.6	4.0
Unidentified preys	13.8	11.4	16.3	14.7	3.3	1.0	1.8	0.3	6.1	1.4	2.6	0.8

Juvenile and maturing size classes of mpun based their diet mainly on crustacea (*%IRI* = 94.1 and 50.6 respectively) with *Pagurus prideauxi* (juveniles: *%F* = 32.4 and *%IRI* = 68.4; maturing: *%F* = 14.8 and *%IRI* = 41.4) as dominant prey (Table 3; Fig. 4). The importance of cephalopoda and fish increased with size with the *%IRI* of the former, increasing from 21.2% to 54.8% in maturing and adult individuals respectively. In this latter size class, *O. vulgaris* was one the most important prey, with approximately 10% of the IRI. Fish prey also increased progressively in mpun stomach contents from juveniles to maturing and adult individuals (*%IRI* = 0.6, 16.1 and 31.4 respectively). In most cases, fish prey identification at the species level was impossible due to the advanced digestion state found (Table 3; Fig. 4).

**Table 3:** Diet composition of the three size classes of *Mustelus punctulatus* %N: percentage in number; %F: Frequency of occurrence; %W: percentage in mass of prey items; %IRI: percentage index of relative importance.

*Prey*	*M. punctulatu*s_juveniles				*M. punctulatus*_maturing				*M. punctulatus*_adults			
	%N	%F	%W	%IRI	%N	%F	%W	%IRI	%N	%F	%W	%IRI
Polychaeta unid	1.2	2.7	0.1	0.1	0.0	0.0	0.0	0.0	0.0	0.0	0.0	0.0
*Chlorotocus crassicornis*	1.2	2.7	0.1	0.1	0.0	0.0	0.0	0.0	0.0	0.0	0.0	0.0
Crustacea	78.6	73.0	85.4	9.4	5.4	44.4	25.7	50.6	16.7	1.3	6.8	5.1
***Anomura***												
*Anomura* unid	0.0	0.0	0.0	0.0	4.7	3.7	0.8	1.5	0.0	0.0	0.0	0.0
*Dardanus arrosor*	2.4	5.4	5.7	1.1	0.0	0.0	0.0	0.0	5.6	6.3	4.0	2.1
*Pagurus anachoretus*	1.2	2.7	0.1	0.1	0.0	0.0	0.0	0.0	0.0	0.0	0.0	0.0
*Pagurus prideauxi*	42.9	32.4	38.9	68.4	25.6	14.8	11.3	41.4	0.0	0.0	0.0	0.0
***Brachiura***												
*Liocarcinus corrugatus*	2.4	5.4	3.6	0.8	11.6	3.7	5.6	4.8	0.0	0.0	0.0	0.0
*Liocarcinus depurator*	22.6	16.2	24.7	19.8	0.0	0.0	0.0	0.0	0.0	0.0	0.0	0.0
*Liocarcinus* spp	6.0	8.1	12.4	3.8	0.0	0.0	0.0	0.0	11.1	6.3	2.7	3.1
*Maja squinado*	0.0	0.0	0.0	0.0	2.3	3.7	5.1	2.1	0.0	0.0	0.0	0.0
*Macroripipus tubercolatus*	0.0	0.0	0.0	0.0	2.3	3.7	0.4	0.8	0.0	0.0	0.0	0.0
*Parapenaeus longirostris*	0.0	0.0	0.0	0.0	4.7	3.7	2.1	1.9	0.0	0.0	0.0	0.0
*Squilla mantis*	0.0	0.0	0.0	0.0	2.3	3.7	0.1	0.7	0.0	0.0	0.0	0.0
*Crustacea* unid	1.2	2.7	0.1	0.1	5.6	7.4	0.2	0.1	0.0	0.0	0.0	0.0
Cephalopoda	1.2	2.7	0.5	0.1	14.0	25.9	50.6	21.2	33.3	31.3	65.9	54.8
*Eledone moschata*	1.2	2.7	0.5	0.1	2.3	3.7	2.6	7.9	0.0	0.0	0.0	0.0
*Loligo vulgaris*	0.0	0.0	0.0	0.0	2.3	3.7	13.8	4.52	0.00	0.00	0.00	0.00
*Octopus vulgaris*	0.0	0.0	0.0	0.0	2.3	3.7	1.8	1.17	11.11	6.3	3.8	10.8
*Scaergus unicirrhus*	0.0	0.0	0.0	0.0	2.3	3.7	0.1	0.7	0.00	0.00	0.00	0.00
*Sepia* spp	0.0	0.0	0.0	0.0	2.3	3.7	0.1	0.7	0.0	0.0	0.00	0.00
Cephalopoda unid	0.0	0.0	0.0	0.0	2.3	7.4	8.8	6.2	22.2	25.0	27.9	44.0
Holothuroidea unid	1.2	2.7	0.2	0.1	4.7	7.4	8.3	7.3	0.0	0.0	0.0	0.0
Teleostea	4.8	1.1	3.4	0.6	18.6	18.5	15.1	16.1	16.7	50.0	21.3	31.4
*Ghathophis mystax*	1.2	2.7	1.0	0.2	2.3	3.7	1.6	1.1	0.0	0.0	0.0	0.0
*Lepidotrigla cavillone*	0.0	0.0	0.0	0.0	2.3	3.7	2.4	1.3	0.0	0.0	0.0	0.0
*Ophisurus serpens*	1.2	2.7	106.0	0.2	0.0	0.0	0.0	0.0	0.0	0.0	0.0	0.0
*Sarda sarda*	0.0	0.0	0.0	0.0	2.3	3.7	1.3	0.4	8.6	1.3	8.0	3.5
*Trachurus trachurus*	0.0	0.0	0.0	0.0	0.0	0.0	0.0	0.0	5.6	6.3	0.2	1.3
*Trigla lucerna*	1.2	2.7	0.4	0.1	0.0	0.0	0.0	0.0	0.0	0.0	0.0	0.0
Teleostea unid	1.2	2.7	0.9	0.1	14.0	7.4	9.8	13.3	11.1	31.3	13.1	26.6
Unidentified preys	1.3	8.1	10.5	4.9	7.0	3.7	0.4	2.1	33.3	6.3	6.1	8.7

The diet of mmus and mpun, based on the analysis of stomach content, was significantly different. In more detail, multivariate analysis of variance (PERMANOVA) based on the prey biomass showed significant interspecific differences (Table 4; Fig. 4).

**Table 4:** Results of permutational multivariate analysis of variance (PERMANOVA) on square root transformed data on the biomass of diet composition of the *M. mustelus* and *M. punctulatus.*

Source	*df*	SS	MS	Pseudo-F	P(perm)	perms
Sp	1	372.13	372.13	6.7918	**0.0001**	9,922
Res	273	14,958	54.791			
Total	274	15,330				

Bold values denote statistical significance.

### Stable isotope values, trophic niche and position

**Figure 5:**
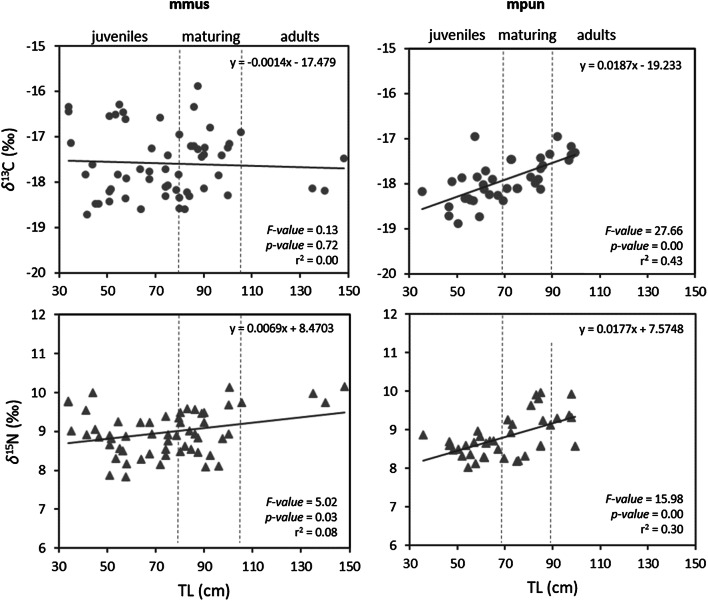
Relationship between the size (total length TL, cm) of the two species of *Mustelus* (mmus and mpun) and the two isotopic signatures *δ*^13^C (‰) and *δ*^15^N (‰). This figure was created using Microsoft Excel by CA.

Isotopic values showed different patterns between the two *Mustelus* species, with *δ*^13^C overall being similar across the three mmus size classes while showing a significantly positive relationship with size in mpun (Fig. 5, *F-value* = 19.78, r^2^ = 0.35, *p-value* < 0.001). The linear regressions between *δ*^15^N and size showed a significantly positive relationship in both species, gradually increasing from juveniles to adult specimens (Fig. 5, mmus: *F-value* = 5.02, r^2^ = 0.08, *p-value* < 0.05; mpun: *F-value* = 15.98, r^2^ = 0.30, *p-value* < 0.001).

The mean trophic position calculated from isotopic data (TP_SIA_) was comprised between about 3.7 and 4.1 in mmus and between 3.6 and 3.9 in mpun (Table 5). Trophic position based on stomach content data (TP_SCA_) showed lower variability among size classes, than TP_SIA_, being 3.7 in all the mmus size classes and varying between 3.4 and 3.6 in mpun (Table 6). Moreover, it should be noted that TP_SIA_ showed a gradual increase across size classes, which was not observed in TP_SCA_.

**Table 5:** Summarized stable isotope data (mean ± standard deviation of *δ*^13^C and *δ*^15^N) for *M. mustelus* and *M. punctulatus* size classes.

Species	Size class	n_SIA_	*δ*^13^C (‰)		*δ*^15^N (‰)		TP_SIA_		n_SCA_	TP_SCA_
			m	SD	m	SD	m	SD		
*M. mustelus*	Juveniles	18	− 17.6	0.9	8.9	0.5	3.7	0.2	72	3.7
	Maturing	36	− 17.4	0.7	9.0	0.6	3.8	0.2	40	3.7
	Adults	4	− 17.7	0.6	9.9	0.2	4.1	0.1	61	3.7
*M. punctulatus*	Juveniles	14	− 18.2	0.4	8.5	0.2	3.6	0.1	33	3.6
	Maturing	20	− 17.6	0.6	9.1	0.6	3.8	0.2	20	3.6
	Adults	5	− 17.36	0.2	9.3	0.5	3.9	0.1	10	3.4

Mean ± standard deviation of the trophic position based on stable isotope data (TP_SIA_) is also shown along with trophic position based on stomach content analysis (TP_SCA_). The number of the individuals taken into account for both methods is also specified.

**Table 6:** Pairwise estimates of the area of niche overlap (‰^2^) between SEAc values of *M. mustelus* (MMUS) and *M. punctulatus* (MPUN) across different size classes: juvenile (juv), maturing (mat) and adults (ad) and mutual % of overlap.

Group 1 versus group 2	SEAc 1	SEAc 2	Overlap	% of SEAc 1 overlapped by SEAc 2	% of SEAc 2 overlapped by SEAc 1
MMUS juv versus MMUS mat	1.5	1.3	1.1	76	83
MMUS juv versus MMUS ad	1.5	0.6	0.0	0	0
MMUS juv versus MPUN juv	1.5	0.3	0.2	12	50
MMUS juv versus MPUN mat	1.5	1.1	09	60	78
MMUS juv versus MPUN ad	1.5	0.4	0.2	15	55
MMUS mat versus MMUN ad	1.3	0.6	0.0	0	0
MMUS mat versus MPUN juv	1.3	0.3	0.0	2	8
MMUS mat versus MPUN mat	1.3	1.1	1.0	72	86
MMUS mat versus MPUN ad	1.3	0.4	0.3	22	77
MMUS ad versus MPUN juv	0.6	0.3	0.0	0	0
MMUS ad versus MPUN mat	0.6	1.1	0.0	0	0
MMUS ad versus MPUN ad	0.6	0.4	0.0	6	8
MPUN juv versus MPUN mat	0.3	1.1	0.0	3	1
MPUN juv versus MPUN ad	0.3	0.4	0.0	0	0
MPUN mat versus MPUN ad	1.1	0.4	0.2	19	54

**Figure 6:**
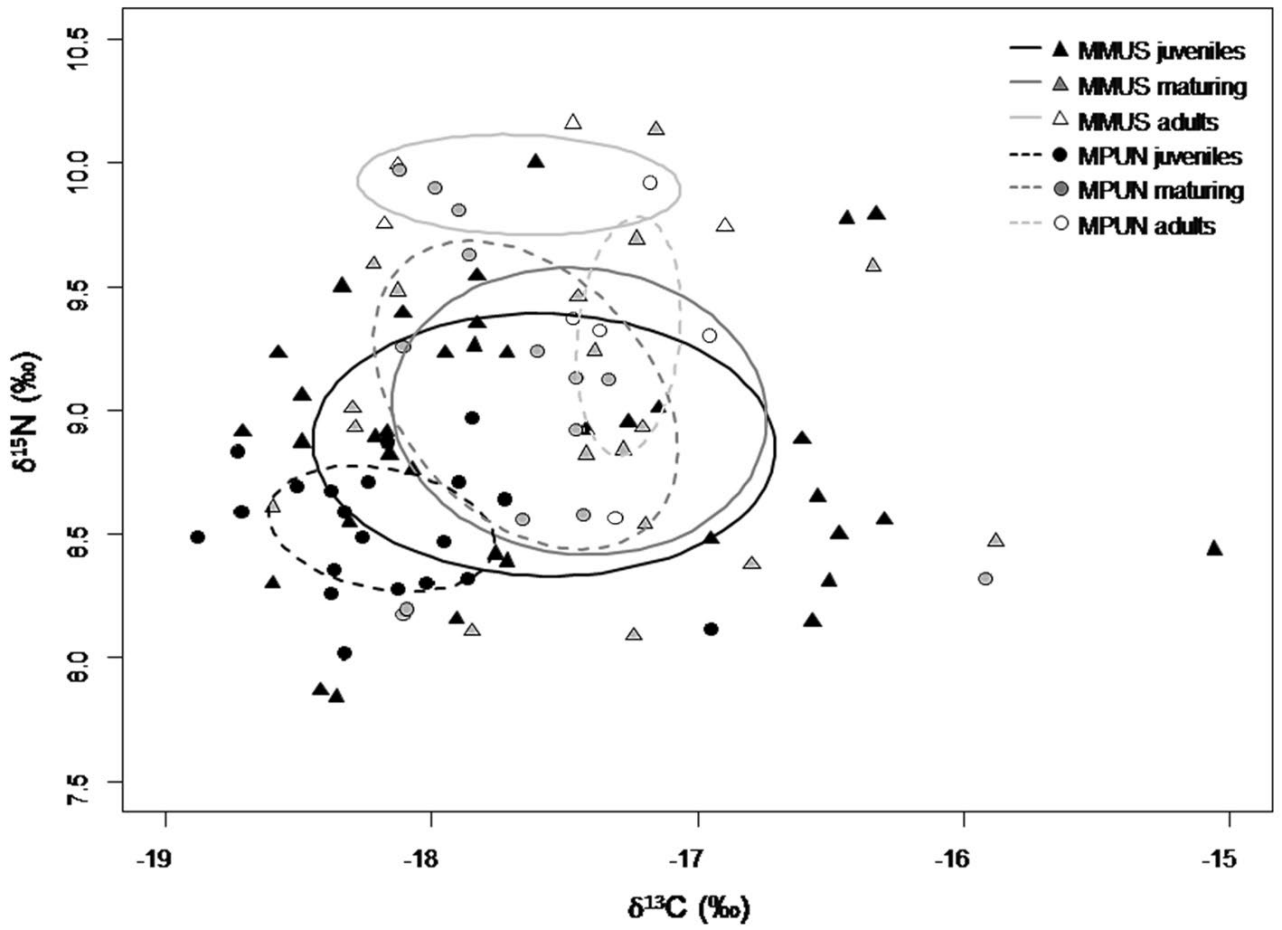
*δ*^13^C (‰) versus *δ*^15^N (‰) of *M. mustelus* (mmus, triangle) and *M. punctulatus* (mpun, circle). Colours correspond to the different size classes: juveniles (black), maturing (grey), adults (white) The isotopic niche of each species and size class is represented by the corrected Standard Ellipse Areas (SEAc) for *M. mustelus* (solid line) and *M. punctulatus* (dashed line) enclosing 40% of the data. This figure was created using R version 3.6.2 https://www.r-project.org/ by CA.

**Figure 7:**
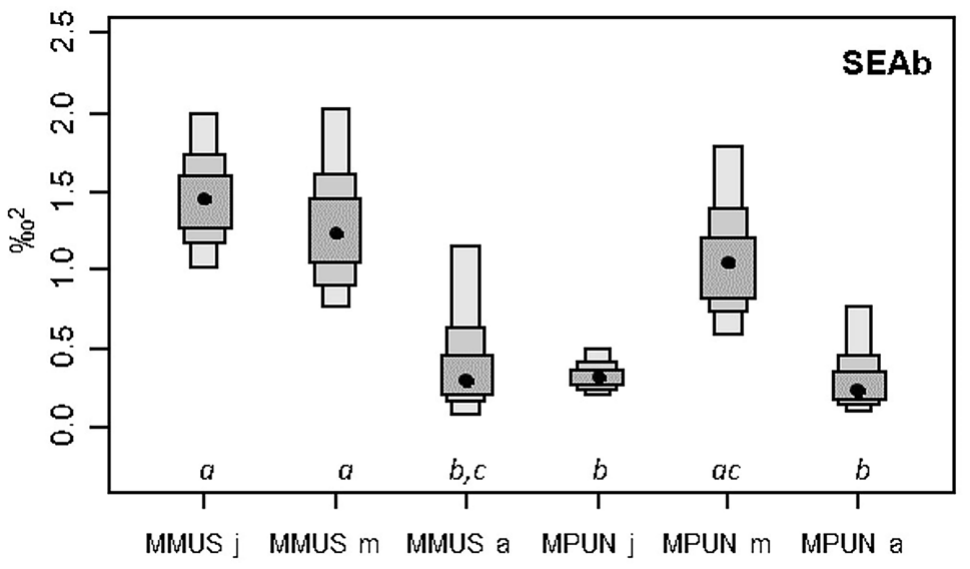
Comparison of estimated Bayesian SEA (SEAb) across different size classes of *M. mustelus* (mmus) and *M. punctulatus* (mpun): j, juveniles; m, maturing; a, adults. Boxes from dark to light grey represent the 50%, 75% and 95% respectively of credibility intervals while black points represent the mode. Italic letters underneath the boxes indicate homogeneous groups based on pairwise probability test (not significant differences for *p* < 095 and *p* > 005). This figure was created using R version 3.6.2 https://www.r-project.org/ by CA.

The isotopic niche of both species and all size classes is represented by the corrected Standard Ellipse Areas (SEAc, Fig. 6). Juveniles of mmus and mpun showed the widest and the narrowest isotopic niche respectively, and only a partial mutual overlap (accounting respectively for the 12% and the 50%, Fig. 6, Table 6). In contrast, the isotopic niche of mmus and mpun maturing specimens showed a high mutual overlap (respectively 72% and 86%), and both were highly overlapped with the niche of mmus juveniles (respectively 83% and 78%). Adult specimens of both species showed a rather narrow niche with a very low reciprocal overlap (respectively 6% and 8%), which was due to the higher δ^15^N values of mmus adults. Only the niche of mpun adults was partially overlapped with the other niches, accounting for about 62% on average (Fig. 6, Table 6). Accordingly, the patterns highlighted by the Bayesian standard ellipse area (SEAb) showed significantly higher values of the isotopic niche width in the mmus juveniles and the maturing specimens of both mmus and mpun, than in the mpun juveniles and the adult specimens of both species, which, in turn, showed the lowest values of the isotopic niche width (Fig. 7).

## Discussion

In this study we compared the diet composition, and, for the first time in the Mediterranean Sea, isotopic niches and trophic positions of two threatened smooth-hounds*,* mmus and mpun, co-occurring in the southern coasts of Sicily (northern sector of the Strait of Sicily, central Mediterranean Sea), across size classes. The study adopted an integrated approach based on the analyses of both stomach contents and stable isotopes. Integrating the two sources of data can be extremely beneficial in studies addressing the trophic ecology of poorly abundant or rare species, where the available number of samples is limited, as for the two smooth-hound of the South of Sicily. Gut content analysis, although useful to determine the composition of the diet, reflects the identifiable food ingested recently, and hence it can underestimate the prey ingested over a longer period. In contrast, stable isotope analysis integrates diet and food assimilation over time (3–4 months, according to^63,68,69^). Although the different time scale of the two approaches may lead to apparently inconsistent results, the combination of both provides complementary insights that may help in disentangling complex trophic aspects. The analyses of stomach contents revealed that the diet of the two smooth-hound species was composed of benthic and demersal species, mostly crabs, cephalopods and fish, showing that they actively search for their prey close to the bottom and then progressively shift toward bigger-sized species during ontogeny. The diet of mmus showed progressive changes during ontogeny, although not so straightforward, with smaller individuals preying mainly on benthic crustaceans (both anomura and brachiura) and fish. The maturing specimens showed a higher reliance on fish, despite consumption of crustaceans remaining fairly high, and the largest specimens relied on big-sized brachiura (i.e. *C. granulata*), fish and cephalopods (e.g. *O. vulgaris*). Prey preference of mpun across size classes showed the juveniles feeding mostly on small hermit crabs (*P. prideauxi*), maturing specimens broadened their diet to include fish and cephalopods that became the almost exclusive prey of adults. These findings highlight that ontogenetic shift in diet was more evident for mpun*,* compared with the congeneric mmus, consistent with previous studies carried out in Tunisia^25,40^ and the Adriatic Sea^37,39^.

A similar pattern in dietary shift during growth was found for mmus in South Africa^70^ and confirms that a diet mostly based on crustaceans, mainly anomura, often with fish and cephalopods as secondary prey is a common feature in smooth-hounds, and observed in many different species and marine regions (see^71^ and references therein).

Nevertheless, unlike our findings, along the Tunisian coasts, the two species showed a wider trophic spectrum with mmus preying upon several species of crustaceans, fish and cephalopods, along with sipunculids, polychaetes and echinoderms as occasional prey^40^ and a similar pattern was highlighted for mpun^25^. Dietary studies on mpun from the Adriatic Sea also reported the occurrence of crustaceans, fish and cephalopods in their stomachs^37,39^ with an increased predation of molluscs in adult sized individuals, as already observed in Tunisian waters^25,40^. More similar to our findings, the only study carried out in the Western Mediterranean (Gulf of Valencia, Spain) reported that the common smooth-hound juveniles (TL < 75 cm) prey mostly upon *Liocarcinus* spp. crabs and the stomatopoda *Squilla mantis*^72^. Similarly, a study in the Aegean Sea identified crustaceans as the main prey of mmus^73^. Fish, instead, was the main prey of *Mustelus mustelus* in Lybian waters^74^.

The analyses of the isotopic niches of the two congeneric *Mustelus* species across size classes well mirrored the results obtained through the stomach contents analysis. Particularly, consistent with the differences in diet composition between the juvenile specimens of the two species, the related isotopic niches were only partially overlapped, with the niche width of mmus wider than that one of mpun*.* This result mirrors the wider carbon and nitrogen ranges of the former species, compared with the latter, and is most probably driven by the higher diversity of prey consumed (26 vs. 15 prey items for mmus and mpun respectively). Besides the two common dominant prey (*P. prideauxi* and *L. depurator*), other prey showed a high relative importance (> 5%) in juvenile mmus*,* among which other anomura, fish and unidentified organic remains, differing from mpun. In contrast to juveniles, maturing specimens showed wide and highly overlapped niches as well as similar isotopic values. Although in contrast with diet composition results, this isotopic similarity reflects a trophic switch observed in both species that, besides crustaceans, started to include bigger sized-prey with a higher trophic level in their diet, such as cephalopods and fish^37,39,75^. For example, maturing mpun specimens*,* showed a niche much wider than the one of juveniles and was coupled by an increase of the *δ*^15^N values, confirming the gradual ontogenetic shift toward prey at a higher trophic level (e.g. fish and cephalopods). The niche expansion was followed by a further narrowing, that was evident in the adult specimens of both species and could be due to species-specific trophic habits on a few prey items, that also implies a clear niche segregation^75,76^. The low number of adult samples however does not allow to exclude a sample-size effect on the observed reduction in prey diversity and further studies are required to better explore this pattern.

The high *δ*^15^N values recorded in adult specimens are indeed consistent with predation on carnivorous prey, such as cephalopods and fish, but seem less consistent with the almost exclusive dominance of the crab *C. granulata* in the mmus stomachs. Although very poor information is available so far about the diet of *C. granulata* (bivalves in^77^; crab remains from author personal observations), the shovel-shaped chelae allow Calappidae crabs to break hard shells and carapaces revealing a carnivorous opportunistic foraging behaviour^78^ that may imply also a high trophic level. It is worth noting that probably the segregation between adults of both species is not only due to the segregation in diet but also to difference in body sizes since mmus adults are almost 20 cm longer than mpun adults as shown in Fig. 5. The low trophic niche overlap, especially between the juveniles and adults of both species, highlighted here, suggests that resource partitioning facilitates coexistence. This is particularly important for endangered species living in the same area and competing for the same resources in their different ontogenetic stages^79^. Such information may provide useful insights about dietary changes occurring during ontogeny and may strongly improve conservation strategies.

Trophic position based on stable isotope (TP_SIA_) and stomach content (TP_SCA_) data of the two shark species was overall comparable in most cases, but for adults TP_SCA_ values were slightly lower compared to TP_SIA_. Moreover, the patterns of trophic position across size classes were different: consistently with isotopic niche patterns, isotopic values and TP_SIA_ subtly increased across size classes, especially in mpun, also revealing the highest values in adult mmus, while TP_SCA_ did not. Slight variations between the two methods have already been observed in^80^ and^81^ and have been linked to the intrinsic differences of the methodological approaches.

Although the mean trophic position of both species was overall lower than previous results for maturing specimens of *Mustelus* spp. (4.0 ± 0.3 in^64^, 3.9 and 4.3 in^65^, 4.2 ± 0.6 in^58^), ontogenetic diet shifts are common features in sharks and mirror an increased preference toward prey at higher trophic levels^76,82,83^. The observed progressive diet shift of the two smooth-hounds, occurring with a reduction in their interspecific trophic overlap during growth, can play an important role in promoting species coexistence, as shown also in other studies^84,85^. Lack of a strong interspecific segregation, as expected for two ecological and morphological similar species, such as mmus and mpun, can be the effect of a reduced trophic competition. Past and current overexploitation has strongly reduced the size of the *Mustelus* populations in the Mediterranean region, thus reducing any selective pressure that would have induced an interspecific shift in prey preferences. Nevertheless, field studies on Mediterranean marine fish have shown that coexistence of trophic guilds (i.e. group of species with similar trophic preferences) is promoted by species segregation along the depth/habitat dimension, so that species with similar prey preference are generally distributed in different habitats^86^. In the case of the two shark species, a lack of a significant segregation both along the habitat and trophic habits can be an indication that competition is not a critical factor for species coexistence. In the literature, several mechanisms have been described to minimize competition for food resources, such as prey size, species segregation, habitat separation, differentiation in foraging tactics, predator size and morphology^87,88^. Among these mechanisms, a differentiation in foraging tactics could partially explain the results obtained in this study.^85,89^.

It is worth noting that one of the main limits of our work is the sample size particularly for adults, as well as the lack of data on interannual changes in diet composition cannot allow to deeply explore interspecific differences in feeding strategy and how these can promote species coexistence. The results of this study however can reflect the current depleted status of the *Mustelus* populations where the selective pressure for segregation in diet between the two species is presumably low. Further studies in this direction should be carried out especially if sound conservation measures will be implemented to protect and rebuild *Mustelus* populations in the SoS.

## Conclusions

Although trophic interactions among organisms are one of the crucial drivers of ecosystem dynamics, dietary information of wide-ranging predators is often missing. Still today, the knowledge of the trophic role of demersal sharks in the Mediterranean Sea remains limited to the most common species, such as *Galeus melastomus* and *Etmopterus spinax*^90^, whereas, there is a poor knowledge about the lesser abundant species. Among these, *Mustelus* spp. played a very important role in the nectobenthic communities of the Mediterranean continental shelf, as witnessed by the relative abundance found in old surveys and catch records^29^. The SoS is one of the Mediterranean areas still hosting viable populations of smooth-hounds, probably due to the occurrence of large off-shore banks that offer refugia and protection from fishing^41^. Both smooth-hound species here studied are common in this area, including both African (i.e. Tunisia, Libya) and Italian (Sicily) waters, but they are still highly exploited by targeted artisanal fisheries and show an alarming decline^91^.

With this study, we improved the knowledge of both diet composition and trophic niches of mmus and mpun, two endangered sympatric species showing very similar morphological characteristics and apparently sharing the same habitats^53^.

By applying for the first time the complimentary and robust approaches based on stomach contents and stable isotopes, we found that the two endangered sympatric *Mustelus* species inhabiting the north sector of the Strait of Sicily share similar trophic habits, although showing a progressive interspecific segregation during growth. The main findings highlight a rather generalist feeding behaviour mostly based on benthic crustaceans for the juvenile and maturing specimens of mmus, followed by a trophic niche narrowing in the adult stage. While mpun showed narrow niches during both the juvenile and adult stages, with a progressive ontogenetic shift from benthic crustaceans to cephalopods and fish, interspersed with a niche widening in maturing specimens.

Lastly, this study confirms that trophic shifts during growth play a key role in the foraging ecology of sharks and is a crucial aspect for understanding the ecological role of these predators. Furthermore, by providing quantitative trophic information, this study gives useful insights for defining future conservation strategies in light of the dramatic decline of smooth-hounds in the Mediterranean Sea, by making evident the possible trophic cascading effects that may be induced by the rebuilding of their populations.
